# Adinizer-Processed Microfragmented Adipose Tissue With Ligament-Targeted Prolotherapy for Acute-on-Chronic Kellgren-Lawrence Grade III Knee Osteoarthritis: A Case Report With 22-Month Follow-Up

**DOI:** 10.7759/cureus.110848

**Published:** 2026-06-14

**Authors:** Yonghyun Yoon, Jihyo Hwang, Jaeyoung Lee, Jaewoo Lim, Seungbeom Kim, Jonghyeok Lee, Teinny Suryadi, Anwar Suhaimi, King Hei Stanley Lam

**Affiliations:** 1 Orthopedics, International Academy of Musculoskeletal Medicine, Hongkong, HKG; 2 Orthopedics, International Academy of Regenerative Medicine, Incheon, KOR; 3 Orthopedics, Musculoskeletal Ultrasound (MSKUS), San Diego, USA; 4 Orthopedic Surgery, Hallym University Kangnam Sacred Heart Hospital, Seoul, KOR; 5 Orthopedic Surgery, Incheon Terminal Orthopedic Surgery Clinic, Incheon, KOR; 6 Orthopedics, Incheon Terminal Orthopedic Surgery Clinic, Incheon, KOR; 7 Orthopedic Surgery, Incheon Terminal Orthopedic Surgery Clinic, Incheon , KOR; 8 Pain Medicine, Miso Pain Clinic, Suwon, KOR; 9 Neurosurgery, Bareun Neurosurgery Clinic, Cheongju-si, KOR; 10 Physical Medicine and Rehabilitation, Medistra Hospital, Jakarta, IDN; 11 Physical Medicine and Rehabilitation, Synergy Clinic, Jakarta, IDN; 12 Physical Medicine and Rehabilitation, Hermina Podomoro Hospital, Jakarta, IDN; 13 Rehabilitation Medicine, University Malaya Medical Centre, Kuala Lumpur, MYS; 14 Rehabilitation Medicine, University Malaya, Kuala Lumpur, MYS; 15 Clinical Research, International Academy of Regenerative Medicine, Incheon, KOR; 16 Clinical Research, The Indonesian Malasian Regenerative Institute, Jakarta, IDN; 17 Clinical Research, The International Association of Musculoskeletal Medicine, Kowloon, HKG; 18 Pain Management and Musculoskeletal Medicine, The Chinese University of Hong Kong, New Territories, HKG; 19 Pain Management and Musculoskeletal Medicine, The University of Hong Kong, Hong Kong, HKG; 20 Clinical Research, The Hong Kong Institute of Musculoskeletal Medicine, Kowloon, HKG

**Keywords:** adinizer, knee osteoarthritis, mfat, microfragmented adipose tissue, orthobiologics, regenerative medicine, stem cell treatment

## Abstract

Knee osteoarthritis is increasingly understood as a whole-joint disorder involving not only articular cartilage but also subchondral bone, synovium, menisci, capsule, ligaments, periarticular entheses, and neuromuscular control. In patients with pre-existing degenerative osteoarthritis whose symptoms worsen after an injury-related episode, treatment strategies focused only on the intra-articular cartilage environment may not adequately address coexisting ligamentous or capsular pain generators. Microfragmented adipose tissue has been explored as an autologous orthobiologic option for symptomatic knee osteoarthritis, while dextrose prolotherapy has been used for chronic musculoskeletal pain and ligamentous or capsular pain generators.

We report the case of a 63-year-old woman with symptomatic right knee osteoarthritis who presented with a 12-month history of right knee pain beginning in May 2023, which worsened after a walking-related injury episode. She had osteoporosis, dyslipidemia, gastroesophageal reflux disease, a history of breast cancer surgery, and previous contralateral unicompartmental knee arthroplasty. Baseline radiographs demonstrated Kellgren-Lawrence grade III right knee osteoarthritis. Because she wished to delay arthroplasty due to previous postoperative pain and prolonged rehabilitation after contralateral knee surgery, she underwent intra-articular injection of 10 mL of autologous microfragmented adipose tissue processed by Adinizer (BSL Co. Ltd., Busan, KOR). Activities of daily living were resumed immediately as tolerated. Rehabilitation progressed from hip abductor and adductor isometric exercises to proprioceptive neuromuscular facilitation and band-resistance exercises. Nonsteroidal anti-inflammatory drugs were discontinued during follow-up. At three and six months, adjunctive prolotherapy was performed to clinically selected ligamentous and capsular targets, including the anterior cruciate ligament, posterior cruciate ligament, medial collateral ligament, lateral collateral ligament, and coronary ligament regions. Dextrose prolotherapy was not performed as a general non-targeted intra-articular osteoarthritis injection protocol.

At the latest clinical follow-up, the visual analog scale (VAS) pain score improved from 8 to 2. Western Ontario and McMaster Universities Osteoarthritis Index (WOMAC) subscores improved from 12 to 4 for pain, 6 to 1 for stiffness, and 38 to 11 for physical function. The total WOMAC score improved from 56 to 16. No donor-site pain, swelling, infection, or clinically significant adverse event was observed. Radiographs obtained in March 2026, approximately 22 months after treatment, showed persistent osteoarthritic changes without obvious radiographic progression.

This report should not be interpreted as evidence that this combined intervention is effective for Kellgren-Lawrence grade III knee osteoarthritis in general or as support for a standardized treatment protocol. Rather, it describes a highly selected, arthroplasty-averse patient in whom a staged, individualized orthobiologic approach supplemented by clinically guided periarticular and capsuloligamentous prolotherapy was associated with sustained symptomatic improvement.

## Introduction

Knee osteoarthritis is one of the most common causes of pain and disability in older adults. Although it has traditionally been discussed in relation to articular cartilage degeneration, current understanding emphasizes osteoarthritis as a disease of the entire joint organ, involving cartilage, subchondral bone, synovium, menisci, capsule, ligaments, periarticular entheses, and altered neuromuscular control [1,2]. This concept is clinically important because pain severity and functional limitation do not always correlate directly with radiographic cartilage loss alone.

A further challenge is that many patients with established degenerative osteoarthritis experience symptom worsening after a specific injury-related event. In such cases, the clinical presentation may represent an acute-on-chronic pattern, in which pre-existing osteoarthritic changes are compounded by trauma-associated inflammation, capsuloligamentous irritation, clinically suspected meniscal pain contribution, subchondral bone stress, or altered joint mechanics [3,4]. Therefore, a treatment strategy aimed only at the intra-articular cartilage environment may be incomplete when ligamentous, capsular, or periarticular pain generators are also clinically suspected.

Autologous orthobiologic therapies have gained interest as joint-preserving options for patients who wish to delay or avoid arthroplasty. Microfragmented adipose tissue is a mechanically processed adipose-derived tissue product that retains stromal vascular elements and extracellular matrix components. Although often discussed by patients as a 'stem cell' treatment, microfragmented adipose tissue is more accurately described as a minimally manipulated, mechanically processed adipose-derived tissue product rather than a purified or culture-expanded stem cell therapy [5-9]. Prior clinical studies have reported symptomatic improvement after microfragmented adipose tissue injection for knee osteoarthritis, but the evidence remains heterogeneous, and structural disease modification has not been established [10,11].

Dextrose prolotherapy has also been investigated in knee osteoarthritis and chronic musculoskeletal pain. Previous knee osteoarthritis prolotherapy protocols have commonly included both intra-articular and extra-articular or periarticular injections [12,13]. In the present case, however, intra-articular treatment was performed using microfragmented adipose tissue prepared by Adinizer (BSL Co. Ltd., Busan, KOR), while dextrose prolotherapy was subsequently restricted to ligamentous and capsular structures. This was intended to separate the intra-articular orthobiologic target from the ligamentous and periarticular pain-generator target as much as possible within an individualized, hypothesis-generating clinical approach.

Here, we report a case of acute-on-chronic Kellgren-Lawrence grade III knee osteoarthritis in a patient with right knee pain beginning in May 2023 and symptom aggravation after a walking-related injury episode, treated with intra-articular Adinizer-prepared microfragmented adipose tissue, staged rehabilitation, and delayed ligament-targeted prolotherapy, with 22-month clinical and radiographic follow-up. This report is intended to describe the clinical reasoning, staged treatment sequence, and follow-up observations in a single selected patient, rather than to establish efficacy or propose a standardized treatment protocol.

## Case presentation

A 63-year-old woman presented in May 2024 with a 12-month history of right knee pain beginning in May 2023. She reported that her symptoms had worsened after a prior injury-associated episode during walking and that she had been told elsewhere that a meniscal injury was possible. However, the corresponding MRI images or report was not available for review, and a meniscal tear was not objectively confirmed in our clinic. Her medical history included osteoporosis, dyslipidemia, and gastroesophageal reflux disease. She also had a history of breast cancer surgery. Orthopedic history was notable for prior left unicompartmental knee arthroplasty for medial compartment pathology.

The patient reported pain during stair descent and pain when standing after prolonged sitting. She also described morning stiffness and discomfort during standing and walking. On physical examination, there was no clinically apparent knee effusion, and the right knee demonstrated a nearly full range of motion. The McMurray test reproduced the patient’s familiar pain, raising clinical suspicion of a possible meniscal pain contribution but not confirming a meniscal tear. Posterior drawer and valgus stress testing also reproduced pain, but these findings were not interpreted as objective evidence of ligament tear or instability. The patellar grind test was negative. These findings supported a symptomatic medial compartment osteoarthritis pattern with clinically suspected contributions from meniscal, capsuloligamentous, or capsular pain, rather than an isolated patellofemoral pain pattern. However, these clinical findings should be interpreted as pain-provocation findings rather than objective structural diagnoses. Although the pain was not yet severe enough for her to immediately proceed with surgical treatment, she strongly wished to delay or avoid right knee arthroplasty because of her previous experience of postoperative pain and prolonged rehabilitation after contralateral knee surgery.

**Figure 1:**
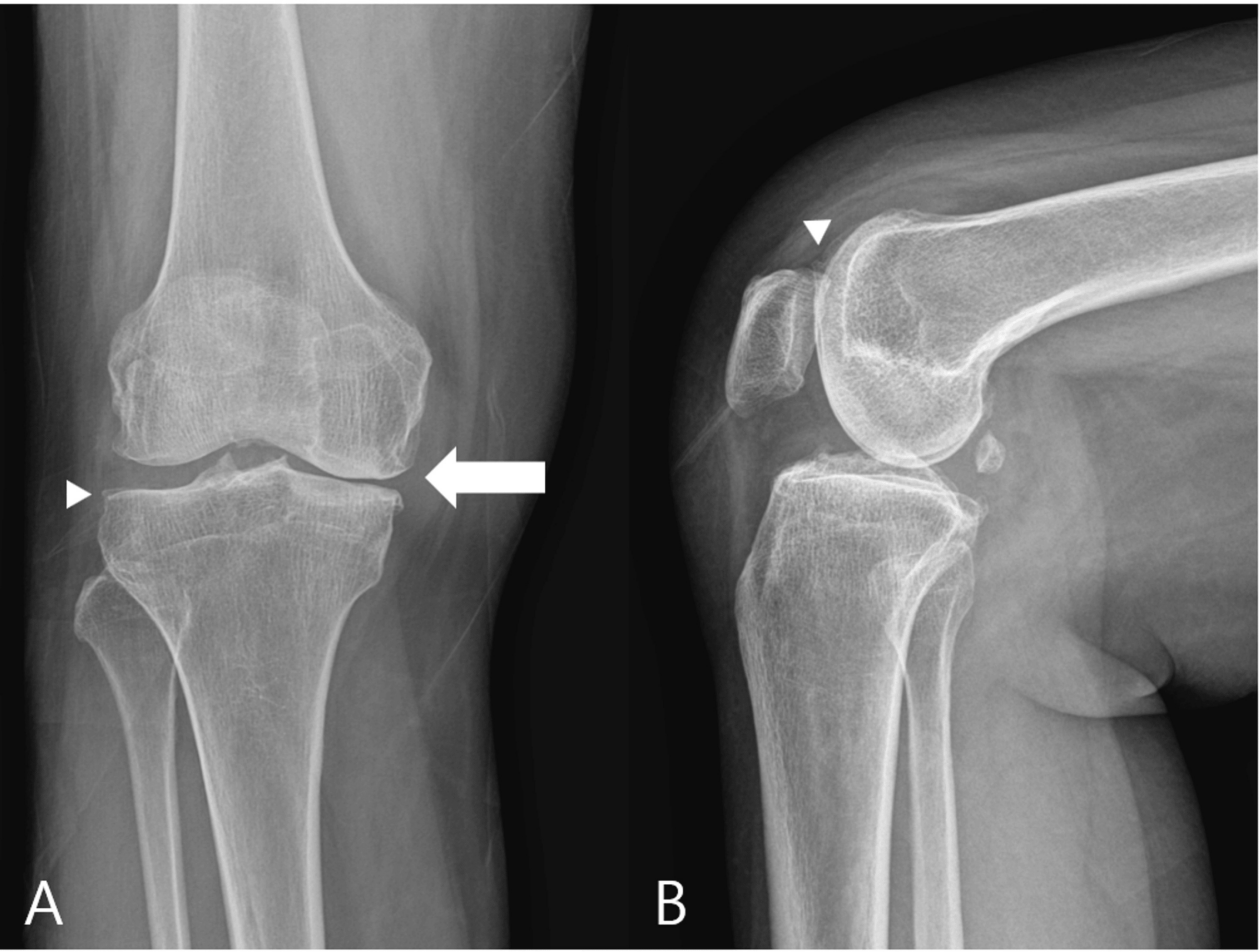
Baseline radiographic findings of the right knee from the initial visit A: The anteroposterior radiograph demonstrates medial compartment joint-space narrowing, marginal osteophyte formation, and degenerative changes consistent with Kellgren-Lawrence grade III osteoarthritis. The white arrow indicates medial compartment joint-space narrowing, and the white arrowhead indicates marginal osteophyte formation. B: The lateral radiograph demonstrates degenerative osteoarthritic changes, including patellofemoral osteophyte formation, indicated by the white arrowhead.

Plain radiographs of the right knee obtained at presentation demonstrated medial compartment joint-space narrowing, marginal osteophyte formation, and degenerative changes consistent with Kellgren-Lawrence grade III osteoarthritis (Figure 1, panels A and B) [14]. Based on the chronic degenerative radiographic findings, injury-associated symptom aggravation, functional pain pattern, and patient preference for a joint-preserving option, a staged joint-preserving treatment plan was developed.

Autologous adipose tissue was harvested from the abdomen. After standard sterile preparation, tumescent solution was infiltrated into both abdominal donor areas, with approximately 150 mL injected into each side. Liposuction was then performed to obtain lipoaspirate. The harvested adipose tissue was washed and mechanically processed using the Adinizer system. After processing, approximately 10 mL of microfragmented adipose tissue was obtained and injected intra-articularly into the right knee.

Following the procedure, the patient was allowed to resume activities of daily living immediately as tolerated. A staged rehabilitation program was initiated. The program began with hip abductor and adductor isometric exercises, progressed to proprioceptive neuromuscular facilitation exercises, and then advanced to band-resistance exercises. Nonsteroidal anti-inflammatory drugs and other anti-inflammatory medications were discontinued throughout the follow-up period according to the treating clinician’s post-injection protocol, while acknowledging that evidence supporting an optimal medication restriction period remains uncertain.

The patient was initially followed at monthly intervals for the first three months and then at three-month intervals. At three and six months after the intra-articular microfragmented adipose tissue injection, adjunctive prolotherapy was performed under ultrasound guidance around clinically selected ligamentous and capsular attachment sites. The injectate consisted of a final mixture of 10% dextrose and 0.2% lidocaine, and approximately 0.2 mL was injected at each attachment point. The target regions included the anterior cruciate ligament, posterior cruciate ligament, medial collateral ligament, lateral collateral ligament, and coronary ligament regions as clinically selected capsuloligamentous attachment sites. These targets were selected based on the patient’s residual symptom pattern and pain-provocation findings, not on MRI-confirmed ligamentous lesions. This was not performed as a generalized or standardized intra-articular dextrose protocol for knee osteoarthritis but as an individualized ligament- and capsule-targeted adjunct based on the patient’s pain pattern and physical examination findings. No additional non-targeted intra-articular dextrose prolotherapy was performed.

**Figure 2:**
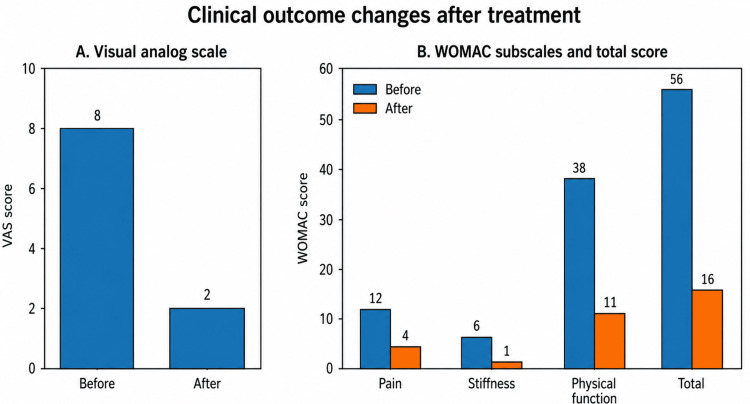
Clinical outcome changes from baseline to the latest clinical follow-up The VAS pain score decreased from 8 at baseline to 2 at the latest clinical follow-up. The WOMAC pain score improved from 12 to 4, stiffness from 6 to 1, physical function from 38 to 11, and total WOMAC score from 56 to 16. VAS: Visual analog scale, WOMAC: Western Ontario and McMaster Universities Osteoarthritis Index

Clinical outcomes improved progressively during follow-up. The VAS pain score decreased from 8 at baseline to 6 at one month, 3 at three months, 2 at six months, and 2 at the latest clinical follow-up. The WOMAC pain subscore decreased from 12 at baseline to 10 at one month, 5 at three months, 4 at six months, and 4 at the latest clinical follow-up. The WOMAC stiffness subscore decreased from 6 to 4, 2, 1, and 1, respectively, and the WOMAC physical function subscore decreased from 38 to 30, 15, 12, and 11, respectively [15]. The total WOMAC score decreased from 56 at baseline to 44 at one month, 22 at three months, 17 at six months, and 16 at the latest clinical follow-up, corresponding to a 71.4% reduction in total WOMAC symptom burden at the latest follow-up (Figure 2). The treatment course, follow-up schedule, and clinical progression are summarized in Table 1. Serial changes in VAS and WOMAC scores are summarized in Table 2.

**Table 1:** Timeline of treatment, follow-up, and clinical course VAS: Visual analog scale, WOMAC: Western Ontario and McMaster Universities Osteoarthritis Index

Timeline	Clinical course and intervention
Initial visit, May 2024	The patient presented with a 12-month history of right knee pain beginning in May 2023, which had worsened after a prior injury-associated episode. Baseline radiographs demonstrated Kellgren-Lawrence grade III osteoarthritis. The baseline VAS pain score was 8. The baseline WOMAC pain, stiffness, physical function, and total scores were 12, 6, 38, and 56, respectively.
Procedure	Autologous abdominal adipose tissue was harvested, washed, and mechanically processed using the Adinizer system. Approximately 10 mL of microfragmented adipose tissue was injected intra-articularly into the right knee.
Immediate post-procedure period	Activities of daily living were resumed immediately as tolerated. A staged rehabilitation program was initiated, beginning with hip abductor and adductor isometric exercises, followed by proprioceptive neuromuscular facilitation exercises and band-resistance exercises. Nonsteroidal anti-inflammatory drugs and other anti-inflammatory medications were discontinued during follow-up.
One month after treatment	The VAS pain score decreased from 8 to 6, and the total WOMAC score decreased from 56 to 44.
Three months after treatment	The VAS pain score decreased to 3, and the total WOMAC score decreased to 22. Ligament- and capsular-targeted prolotherapy was performed around the anterior cruciate ligament, posterior cruciate ligament, medial collateral ligament, lateral collateral ligament, and coronary ligament regions.
Six months after treatment	A second session of ligament- and capsular-targeted prolotherapy was performed. The VAS pain score was 2, and the total WOMAC score was 17. Clinical improvement had stabilized by this time point and remained similar to the latest clinical follow-up status.
Six to 22 months after treatment	Symptomatic improvement remained stable without clinically meaningful worsening. The patient continued follow-up at three-month intervals. No donor-site pain, swelling, infection, or clinically significant post-injection inflammatory reaction was observed.
22-month radiographic follow-up, March 2026	Follow-up radiographs demonstrated persistent medial compartment osteoarthritic changes without obvious radiographic progression compared with baseline radiographs obtained in May 2024.
Latest clinical follow-up	The VAS pain score was 2. The WOMAC pain, stiffness, physical function, and total scores were 4, 1, 11, and 16, respectively. The patient reported improved tolerance of stair ascent and descent and improved activities of daily living. She did not undergo right knee arthroplasty during the follow-up period.

**Table 2:** Serial changes in VAS and WOMAC scores during follow-up The WOMAC was scored using the 24-item Likert version, with each item scored from 0 to 4; subscale ranges were 0-20 for pain, 0-8 for stiffness, 0-68 for physical function, and 0-96 for the total score. Lower scores indicate lower symptom burden. VAS: Visual analog scale, WOMAC: Western Ontario and McMaster Universities Osteoarthritis Index

Timeline	VAS pain score	WOMAC pain	WOMAC stiffness	WOMAC physical function	Total WOMAC score
Baseline, May 2024	8	12	6	38	56
One month after treatment	6	10	4	30	44
Three months after treatment	3	5	2	15	22
Six months after treatment	2	4	1	12	17
Latest clinical follow-up	2	4	1	11	16

**Figure 3:**
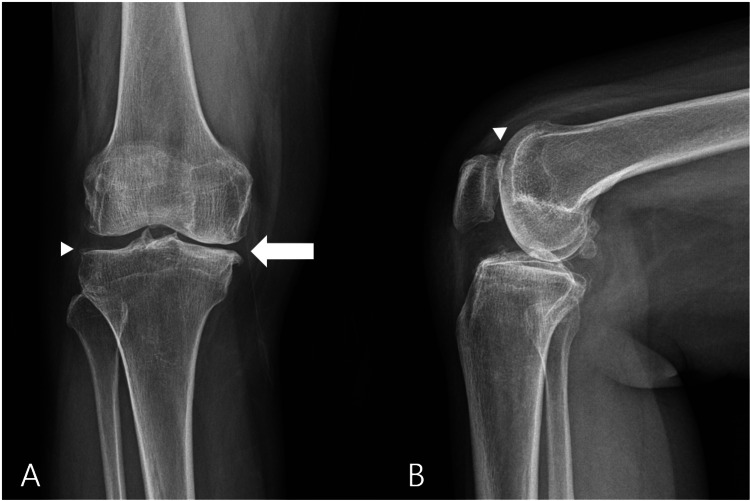
Follow-up radiographic findings of the right knee 22 months after treatment A: The anteroposterior radiograph demonstrates persistent medial compartment joint-space narrowing (white arrow) and marginal osteophyte formation (white arrowhead) without obvious interval progression compared with baseline imaging. B: The lateral radiograph demonstrates persistent degenerative osteoarthritic changes (white arrowhead) without obvious interval progression.

At the latest clinical follow-up, the patient reported improved tolerance for stair ascent and descent and improved activities of daily living. No procedure-related adverse events, including donor-site pain, swelling, infection, or clinically significant post-injection inflammatory reaction, were observed. Adverse events were assessed through patient interviews and routine clinical examinations during follow-up visits; no standardized adverse-event questionnaire was used. Standing anteroposterior and lateral radiographs obtained in March 2026, 22 months after treatment, demonstrated persistent medial compartment osteoarthritic changes without obvious interval progression compared with baseline radiographs obtained in May 2024 (Figure 3, panels A and B). The Kellgren-Lawrence grade remained grade III at both time points. Because the radiographs were obtained as routine clinical images without a calibration marker or standardized fixed-flexion acquisition protocol, absolute millimeter-based joint-space width measurement was not used as a structural outcome. The patient did not undergo right knee arthroplasty during the follow-up period.

## Discussion

This case describes sustained symptomatic improvement after an Adinizer-prepared microfragmented adipose tissue-centered regenerative strategy in a patient with acute-on-chronic Kellgren-Lawrence grade III knee osteoarthritis. Serial clinical assessment demonstrated stepwise improvement, with the VAS pain score decreasing from 8 at baseline to 6 at one month, 3 at three months, 2 at six months, and 2 at the latest clinical follow-up. The total WOMAC score improved from 56 at baseline to 44 at one month, 22 at three months, 17 at six months, and 16 at the latest clinical follow-up. Standing radiographs obtained at the 22-month follow-up showed persistent osteoarthritic changes without obvious interval radiographic progression. These findings should be interpreted as a hypothesis-generating observation in a single selected patient rather than as evidence of generalizable efficacy or structural disease modification.

The main clinical significance of this case is not that it proves disease reversal or cartilage regeneration, but that it illustrates a pragmatic whole-joint treatment strategy in a selected patient with established osteoarthritis and injury-associated symptom aggravation. Osteoarthritis is increasingly viewed as a disease of the joint as an organ rather than a disorder of cartilage alone [2]. This concept is especially relevant in patients whose symptoms worsen after a traumatic or mechanical episode, because post-injury inflammation, capsuloligamentous irritation, meniscal pathology, subchondral bone stress, and altered neuromuscular control may all contribute to pain and functional limitation [3,4].

In the present patient, the history, physical examination, and radiographic findings suggested an acute-on-chronic clinical pattern. The patient had right knee pain beginning in May 2023, with symptom aggravation after a walking-related injury episode, and a patient-reported possibility of meniscal injury raised elsewhere; however, MRI documentation was unavailable. Physical examination demonstrated no clinically apparent effusion and nearly full range of motion, with reproduction of familiar pain during McMurray testing, pain provocation during posterior drawer and valgus stress testing, and a negative patellar grind test. These findings suggested possible meniscal, ligamentous, or capsular pain contributions without a dominant patellofemoral pain pattern. Radiographs demonstrated established medial compartment degenerative osteoarthritis. This combination provided the rationale for addressing the intra-articular osteoarthritic environment and clinically suspected periarticular pain generators. However, because an MRI was not obtained, the presence, type, or severity of meniscal or ligamentous structural pathology could not be confirmed. The intra-articular component was addressed with Adinizer-prepared microfragmented adipose tissue, whereas delayed prolotherapy was reserved for residual clinically suspected ligamentous and capsular pain generators identified from the patient’s baseline physical examination and follow-up symptom pattern.

Microfragmented adipose tissue has been explored as an autologous orthobiologic treatment for knee osteoarthritis. Its proposed mechanism should not be reduced to a simple 'stem cell injection' concept. Mesenchymal stromal cell biology is complex, and minimal criteria for defining multipotent mesenchymal stromal cells were developed for isolated cell populations rather than mechanically processed adipose tissue products [5,6]. Mechanically processed microfragmented adipose tissue may contain adipose-derived stromal elements, perivascular cells, stromal vascular components, extracellular matrix scaffolds, and paracrine signaling factors [7-9]. These features may contribute to modulation of the inflammatory joint microenvironment, nociceptive signaling, and tissue homeostasis, but the precise mechanism of clinical improvement remains uncertain.

The Adinizer system is a mechanical processing platform designed to micronize lipoaspirate without enzymatic digestion. Adinizer-processed minimally manipulated adipose tissue has been described in prior musculoskeletal and spine-related case reports, including disc-driven cervical myelopathy, osteonecrosis of the femoral head, and degenerative lumbar spondylolisthesis [16-18]. However, these reports involved heterogeneous non-knee indications and were framed as hypothesis-generating clinical observations rather than definitive evidence of structural regeneration. In knee osteoarthritis, previous clinical studies of intra-articular microfragmented adipose tissue have reported encouraging but heterogeneous symptomatic outcomes, and structural disease modification has not been established [10,11].

An important distinction in this case is the way prolotherapy was incorporated. Previous knee osteoarthritis prolotherapy studies have often used protocols that included intra-articular dextrose injection, frequently combined with extra-articular or periarticular injections [12,13]. In contrast, the present protocol used microfragmented adipose tissue as the intra-articular orthobiologic intervention and reserved prolotherapy at three and six months for residual symptoms attributed clinically to ligamentous or capsular attachment sites, rather than for generalized intra-articular treatment of osteoarthritis. The subsequent prolotherapy was performed under ultrasound guidance using a final injectate mixture of 10% dextrose and 0.2% lidocaine, with approximately 0.2 mL injected at each attachment point. The target regions included the anterior cruciate ligament, posterior cruciate ligament, medial collateral ligament, lateral collateral ligament, and coronary ligament regions. This distinction is important because repeated intra-articular dextrose prolotherapy after intra-articular microfragmented adipose tissue could have further complicated the interpretation of the intra-articular response. Therefore, the prolotherapy component should be interpreted as a targeted periarticular and capsuloligamentous adjunct rather than as a repeated general intra-articular dextrose osteoarthritis protocol.

The capsuloligamentous adjunctive approach was based on the clinical concept that osteoarthritis pain may arise from multiple pain generators rather than cartilage alone. In this case, however, the suspected periarticular pain generators were identified clinically and should not be interpreted as MRI-confirmed meniscal or ligamentous pathology. In patients with injury-associated worsening, capsuloligamentous structures may contribute to abnormal joint mechanics, altered load transfer, and persistent pain. This rationale is consistent with previous knee osteoarthritis prolotherapy protocols that incorporated periarticular injections [12,13] and with the broader whole-joint concept that periarticular soft tissues may influence pain and function [2-4].

The staged rehabilitation protocol may also have contributed to the favorable outcome. The patient resumed activities of daily living immediately as tolerated, while exercises progressed from hip abductor and adductor isometrics to proprioceptive neuromuscular facilitation and then band-resistance training. This sequence was intended to improve hip and lower-limb neuromuscular control, reduce abnormal loading across the osteoarthritic knee, and improve stair-related function. Because the patient’s pain was particularly prominent during stair descent and sit-to-stand movement, restoring periarticular muscular control may have been clinically relevant.

The patient discontinued nonsteroidal anti-inflammatory drugs during follow-up. This decision was based on the theoretical concern that anti-inflammatory medication may interfere with the intended inflammatory and reparative phases of regenerative injection therapy. However, the optimal medication restriction period after microfragmented adipose tissue injection or prolotherapy remains uncertain. Therefore, this medication strategy should be reported as part of the clinical protocol, but it should not be interpreted as an independently validated requirement for treatment success.

The radiographic findings should be interpreted cautiously. The absence of obvious progression on plain radiographs over the 22-month follow-up period is encouraging, but it does not prove structural disease modification. Although the anteroposterior radiographs were obtained in the standing position, they were routine clinical radiographs and were not acquired using a calibrated fixed-flexion protocol. Therefore, absolute millimeter-based joint-space width measurement was not used as a structural outcome. Plain radiographs are also limited by patient positioning, knee flexion angle, weight-bearing status, beam angle, and projectional variability. Moreover, radiographs cannot directly evaluate cartilage quality, meniscal integrity, synovitis, bone marrow lesions, ligament status, or subtle subchondral changes. Therefore, the imaging findings in this case should not be described as cartilage regeneration or reversal of osteoarthritis. An MRI would be required to evaluate structural changes more precisely.

Safety is another relevant observation. No donor-site pain, swelling, infection, or clinically significant adverse event was observed during follow-up. This supports procedural tolerability in this patient. However, a single case cannot establish general safety. Larger prospective studies are required to evaluate donor-site morbidity, infection risk, post-injection flare reactions, and long-term outcomes after Adinizer-prepared microfragmented adipose tissue injection.

This report has several limitations. First, it describes a single uncontrolled case and cannot establish causality or generalizability. Second, the patient was highly suitable because she had symptomatic Kellgren-Lawrence grade III knee osteoarthritis, wished to delay or avoid arthroplasty, and was willing to comply with close follow-up and staged rehabilitation, introducing substantial selection bias. Therefore, the observed improvement cannot be generalized to all patients with grade III knee osteoarthritis or interpreted as evidence that this approach can replace arthroplasty. Third, the treatment protocol included multiple components, including intra-articular microfragmented adipose tissue injection, rehabilitation, nonsteroidal anti-inflammatory drug discontinuation, and delayed ligament-targeted prolotherapy; therefore, the relative contribution of each component cannot be separated. Fourth, although serial VAS and WOMAC scores were available, placebo response, regression to the mean, natural symptom fluctuation, and rehabilitation effects may have contributed to the observed improvement. Fifth, radiographic assessment was limited to routine plain radiographs without calibrated fixed-flexion joint-space measurement or MRI; therefore, cartilage, meniscal, synovial, bone marrow, and ligamentous changes could not be evaluated. Sixth, the biological composition of the injected microfragmented adipose tissue was not quantified. Finally, although physical examination suggested meniscal, ligamentous, or capsular pain contributions, the absence of objective ultrasound or magnetic resonance documentation of these structures limits interpretation.

Despite these limitations, this case may still be clinically useful because it illustrates a structured, mechanism-based approach in a select patient with moderate-to-advanced knee osteoarthritis who wished to delay arthroplasty. The intervention should not be interpreted as a cartilage-regeneration procedure or as treatment of MRI-confirmed meniscal or ligamentous pathology. Rather, symptomatic improvement was observed after an Adinizer-prepared microfragmented adipose tissue-centered whole-joint strategy, supplemented by rehabilitation and delayed, clinically guided periarticular and capsuloligamentous prolotherapy for residual pain-provocation findings. Because no baseline or follow-up MRI was obtained, the presence, severity, and temporal change of meniscal tears, ligament integrity, cartilage status, synovitis, bone marrow lesions, and subtle subchondral abnormalities could not be objectively assessed. Therefore, any reference to meniscal, ligamentous, capsuloligamentous, or capsular pain contributions in this report should be interpreted as a clinical pain-generator hypothesis rather than an imaging-confirmed structural diagnosis. Future studies should include standardized patient selection, repeated validated outcome measures, baseline and follow-up MRI or other objective imaging protocols, clear separation of intra-articular orthobiologic treatment from periarticular adjunctive procedures, appropriate comparison groups, and longer follow-up to determine whether this staged approach is reproducible and whether it can delay arthroplasty in selected patients.

## Conclusions

This single case should not be interpreted as evidence that Adinizer-processed microfragmented adipose tissue or ligament-targeted prolotherapy is effective for Kellgren-Lawrence grade III knee osteoarthritis in general. The findings are hypothesis-generating and may be useful for illustrating a staged, individualized approach in a select arthroplasty-averse patient, but the absence of MRI prevents objective confirmation of meniscal, ligamentous, or other soft-tissue structural pathology. Further prospective studies with standardized patient selection, repeated validated outcome measures, objective imaging protocols, and appropriate comparison groups are needed.
